# Pennsylvania State Veterinary Medical Association

**Published:** 1896-07

**Authors:** 


### PENNSYLVANIA STATE VETERINARY MEDICAL ASSOCIATION.

PRESIDENT RIDGE announces the following appointments of delegates to the United States Veterinary Medical Associations and other kindred organizations: United States: J. C. Foelker, James T. McAnulty, George B. Jobson. New York: W. Horace Hoskins, C. C. McLean, Robert Formad. Maryland: W. H. Hoskins, W. B. E. Miller, James B. Rayner. New Jersey: L. O. Lusson, F. S. Allen, H. B. Felton. Ohio: J. C. McNeil, James A. Waugh, N. Rectenwald. Keystone: H. J. McClellan, Charles Lintz, James T. McAnulty. Philadelphia Veterinary Medical Association: William Tag, John W. Adams, M. E. Conard. Schuylkill: J. W. Sallade, J. H. Timberman, W. S. Phillips. Lehigh: Otto Noack, Leonard Pearson, Francis Bridge.

RESOLUTIONS ADOPTED BY THE AMERICAN VETERINARY COLLEGE ALUMNI ASSOCIATION ON THE DEATH OF DR. R. A. MCLEAN. WHEREAS, It has pleased God in His infinite wisdom and plans to remove from our midst our fellow-alumnus, Roderick A. McLean, of the Class of 1879; and be it

*Resolved*, In bowing to His supreme will, we acknowledge the loss of one whose name and face had grown familiar and dear to us all, through his interest and work in this Association; and be it further

*Resolved*, That this loss removes from among us one whose strong traits of character, his strong ties of friendship, his earnest and decided convictions, so much to be admired among men, that in paying this just tribute to his memory it is with a hope that we may strive to emulate the same; and be it further

*Resolved*, That a copy of these resolutions be spread upon our minutes, and be sent to the sorrowing parents.

W. Horace Hoskins (Chairman), William H. Lowe, H. D. Hanson, *Committee.*

